# Editorial: The Evolution of Neuropeptides - a Stroll Through the Animal Kingdom: Updates From the Ottawa 2019 ICCPB Symposium and Beyond

**DOI:** 10.3389/fendo.2020.00508

**Published:** 2020-07-28

**Authors:** Klaus H. Hoffmann, Elizabeth A. Williams

**Affiliations:** ^1^Faculty for Biology, Chemistry, and Earth Sciences, University of Bayreuth, Bayreuth, Germany; ^2^College of Life and Environmental Sciences, University of Exeter, Exeter, United Kingdom

**Keywords:** neuropeptides, neuropeptide evolution, neuropeptide signaling, neuropeptide function, cnidarians, arthropods, echinoderms, metazoan

Signaling in the nervous and endocrine systems via neuropeptides is an ancient mechanism found in almost all animals. Active neuropeptides, generated by enzymatic cleavage of larger precursor peptides, play a role as neurotransmitters, neuromodulators, hormones, or growth factors and are involved in the regulation of a huge variety of biological systems. Through binding to their specific G protein-coupled receptors, neuropeptides regulate processes including animal development and growth, feeding, metabolism and digestion, behavior, diuresis and homeostasis, and ecdysis and metamorphosis. The chemical structure of neuropeptides is highly diverse and every peptide may be pleiotropic in function, making the study of neuropeptide signaling a complex yet fascinating subject.

Recent advances in genome/transcriptome sequencing, in concert with mass spectrometry and computational prediction and processing, have enabled the identification of active peptides, neuropeptide precursor proteins, and neuropeptide receptors in species from a growing variety of animal taxa. Also recently, techniques such as RNA interference, morpholino knockdown, and the CRISPR-Cas system for genome editing have been used for specific knockdown of neuropeptide precursor genes and neuropeptide receptors, to evaluate the function of neuropeptide signaling. These technological advancements have provided novel insights into neuropeptide function and evolution.

In August 2019, scientists from around the world gathered in Ottawa (Canada) to discuss all aspects of neuropeptide evolution, from structural diversity to functional condition and potential applications, as part of the 10th International Congress of Comparative Physiology and Biochemistry: mechanisms and evolutionary processes. This Research Topic collects the findings presented at the neuropeptide symposium plus recent associated research, including six original research papers and four review articles. The papers cover a wide range of metazoans, from cnidarians to echinoderms.

The origin of neuropeptide signaling in metazoans is currently a major focus in neuropeptide biology. Three papers in this collection extend our knowledge of neuropeptide diversity in the early branching cnidarians. Analysis of largescale transcriptome resources from several different classes of cnidaria by Koch and Grimmelikhuijzen identify two ancient cnidarian neuropeptide classes, GRFamides and RPRSamides. This study also uncovers the distribution of different neuropeptide classes across largely understudied cnidarians including cubozoans, scyphozoans, staurozoans, and octocorallia. Complementing this study is a review article by Takahashi summarizing the structure and function of Cnidarian neuropeptides, with focus on hydrozoans and hexacorallians. Cnidarian neuropeptides function in a wide range of processes throughout the life cycle, including neuron differentiation, larval locomotion, metamorphosis, muscle contraction, feeding, sensory activity, and reproduction. Like other animal phyla, the cnidarian neuropeptide repertoire includes both novel and conserved neuropeptides. Zang and Nakanishi initiate investigations into cnidarian-specific neuropeptides by mapping expression of RPamide during development in the starlet sea anemone, *Nematostella vectensis*. This study reveals new features of the sea anemone nervous system, including the sensory larval apical organ, and provides insight into how the nervous system is reshaped during metamorphosis from larval to adult form.

Classic studies of neuropeptide structure and function in arthropods such as fruit fly, cockroach, cricket and crab were imperative in establishing the neuropeptide biology field. Here, four original research papers expand our knowledge of panarthropod neuropeptide signaling in diverse directions. Martin et al. characterize the expression of two pigment dispersing hormones (PDFs) and a PDF receptor in a velvet worm (Phylum Onychophora), enlightening the organization and function of PDF signaling, largely known for involvement in the circadian system, in a panarthropod ancestor. The authors also employ receptor deorphanization to uncover the difference in binding specificity of the two PDFs to this G protein-coupled receptor. Bläser and Predel carry out bioinformatic analysis of an impressive 200 insect species from the group Polyneoptera—cockroaches, termites, locusts, and stick insects—to study the evolution of single-copy neuropeptide precursors. These neuropeptides, including ASTC, CCAP, CCHamide, corazonin, elevenin, NPF, proctolin, SIFamide, and sNPF, show a relatively high degree of sequence conservation, although the extent of conservation differs between neuropeptide families. This provides clues regarding the conservation or diversification of function for polyneopteran neuropeptides. Gäde et al. report a mass spectrometry analysis of the presence and structure of adipokinetic hormones (AKHs) in Diptera (flies, mosquitoes) and Mecoptera (scorpion flies). The authors map how changes in single amino acid-increments from an ancestral peptide could have occurred during dipteran evolution to generate existing AKH diversity, providing a finescale look at neuropeptide evolution. Rocco and Paluzzi investigate the expression of glycoprotein hormone subunits GPA2 and GPB5 and their G protein-coupled receptor LGR1 in the mosquito *Aedes aegypti*. The authors find that GPA2/GPB5 heterodimers activate the LGR1 receptor similar to glycoprotein receptor interactions in human and *Drosophila*. Importantly, the authors noted that each subunit may also interact with other unknown proteins to activate different receptors or signaling pathways for different functions. This study contributes to cross-phylum understanding of neuropeptide signaling as glycoprotein hormone is an ancient neurohormone found across metazoa.

Three review articles contribute to bridging our understanding of neuropeptide signaling across different metazoan phyla.Williams compares and contrasts Wamide neuropeptide family signaling across cnidarians and protostomes. Yañez-Guerra and Elphick survey the luqin family, a paralog of tachykinins, in metazoans. Luqins function in regulating feeding, diuresis, egg-laying, locomotion and lifespan. The identification of luqins in a starfish and hemichordate extends this family into deuterostomes. Nässel et al. review the organization and evolution of multi-functional tachykinin precursor peptides across protostomes and deuterostomes. The authors note that a subset of protostome tachykinins have been recruited for use in venom or salivary glands to affect prey. This interesting evolutionary path of neuropeptide recruitment for novel toxins is also recently revealed in *Nematostella vectensis* through discovery of an ShK-like venom component, which causes muscle contractions in *Nematostella* but is toxic to fish larvae (1).

Sincere thanks to the authors, reviewers, and editors for their valuable contributions to this Research Topic, and to the ICCBP Symposium presenters and participants for their enthusiastic participation. We look forward to moving toward practical applications utilizing knowledge of neuropeptide signaling in the development of pest and parasite control agents and novel drugs, the improvement of invertebrate culture for food, and in bio conservation, both on the land and in the sea.

## Author Contributions

All authors listed have made a substantial, direct and intellectual contribution to the work, and approved it for publication.

## Conflict of Interest

The authors declare that the research was conducted in the absence of any commercial or financial relationships that could be construed as a potential conflict of interest.
